# Mesiodistal crown diameters and tooth size discrepancy of permanent dentition in thalassemic patients

**DOI:** 10.4317/jced.51214

**Published:** 2013-12-01

**Authors:** Faiez N. Hattab

**Affiliations:** 1 BDS, PhD, Odont. Dr. Professor and Senior Consultant in Restorative and Pediatric Dentistry, private clinic. Amman, Jordan

## Abstract

Objectives: To provide a description of mesiodistal crown diameters (MD) and tooth-size discrepancy (TSD) of the permanent dentition in patients with thalassemia major (TM) and to compare the results with those of unaffected control group. Study design: The sample consisted of 46 thalassemic patients, 25 males and 21 females aged 7.3 to 23.7 years (mean ± SD = 11.2 ± 3.9 years) and control group matched by age and sex. Dental casts of the participants were measured for MD, using a digital sliding caliper. Descriptive statistics were computed for each individual tooth. Student t-test was used for comparison of mean values between males and females as well as between thalassemic and control groups. The differences between sets of data were evaluated by analysis of variance (ANOVA). Results: All means for MD of thalassemic males and females were smaller than their controls, with 20 of the 24 comparisons being statistically significant (ranged from P<0.05 to P<0.001). In both thalassemic and control groups, males exhibited significantly larger MD than females in most instances. Canines displayed the most sexual dimorphic teeth in the dentition. Lateral incisors showed the greatest variability indicated by the coefficient of variation, while the first molars were the least variable teeth (7.6% vs. 5.3%). There were no statistically significant differences in the anterior and overall tooth-size discrepancy ratios between sexes or between thalassemic and control groups. The mean anterior ratio (79.5%) and overall ratio (92.4%) of the control group were significantly larger than of Bolton ratios; P< 0.001 and P<0.05, respectively. Conclusions: The present study demonstrated that thalassemic males and females exhibited significantly smaller MD than the control group. The TSD ratios in both thalassemic and control groups were significantly larger than those of Bolton sample. These findings should be taken into account when planning orthodontic treatment for thalassemic patients.

** Key words:**Permanent dentition, tooth size, thalassemia major.

## Introduction

Mesiodistal crown diameter (MD); also called tooth size, tooth crown size, or tooth width, in human populations has been the subject of numerous studies because of its application in human evolution and biological problems as well as in forensic investigations and clinical dentistry. Of clinical dental interest is the interrelation between MD and arch alignment in which large teeth are associated with dental crowding, the most common type of malocclusion (1). A relationship has been demonstrated between tooth size and third molar eruption and impaction (2,3). A correct MD relationship between maxillary and mandibular teeth is important for the achievement of proper interdigitation, overjet, and overbite during the final stages of orthodontic treatment. The well-known study of tooth-size discrepancy (TSD) in relation to treatment of malocclusion was reported by Bolton (4).

Thalassemia is a group of inherited defects in the synthesis of either α- or β-polypeptide chains of hemoglobin, leading to decrease hemoglobin production and hypochromic microcytic anemia. Because of this defect, the condition is referred to as α- or β-thalassemia, with several subtypes manifested in diverse clinical pictures. Due to genetic heterogeneity and clinical and hematological variability, thalassemia is classified as homozygous, heterozygous, or compound heterozygous. The homozygous type of β-thalassemia (also known as thalassemia major, Cooley’s anemia or Mediterranean anemia) exhibits the most severe clinical symptoms; often described as transfusion-dependent disorder.

Thalassemia is one of the most common genetic disorders worldwide, presenting major public health and social problems in the high incidence areas. About 3% of the world’s population carries β-thalassemia gene (5). The disorder is most common among individuals of Mediterranean descent, particularly those living southern Italy, Greece and Cyprus with prevalence 10 to 15%. The condition is also described in Arab countries, Turkey, Iran, Southeast Asia and Africa with frequency ranged 1.5 to 5% of the population. In Jordan, one-third of the country thalassemic patients reside in northern district, where this study was conducted.

Thalassemia major (TM) is life threating condition that commonly manifested during early infancy, after which progressively pallor, severely anemic and failure to thrive are common. Children with TM often develop feeding problems, recurrent fever, susceptibility to infection, pathological fractures of long bones and vertebrae, osteoporosis, endocrine abnormalities, and growth retardation (5,6). Hemoglobin level may be as low as 3 to 5 g/dL when a child with TM become symptomatic. To treat anemic hypoxia, people with TM usually require blood transfusion in order to normalize hemoglobin level.

Skeletal and craniofacial deformities are the common manifestation of TM. They result primarily from hypertrophy and expansion of erythroid marrow due to ineffective erythropoiesis (formation of erythrocytes). The most striking orofacial changes in TM include prominent frontal bossing and cheek bones, overgrowth of the maxilla, flaring of the maxillary anterior teeth and malocclusion (7-9). TM patients are at high risk of dental caries (10,11) and periodontal disease (9). They also have reduced dental arches dimensions (12) and delay in dental development, short stature and underweight (13).

Detailed data on odontometrics and their clinical applications in thalassemic subjects are lacking. The aim of this study, therefore, was to determine the MD and TSD in Jordanian patients with TM and to compare the results with unaffected (thalassemia-free) control group.

## Material and Methods

The sample comprised of 45 patients with TM, 25 males and 20 females aged 7.3 to 18.3 years, with the mean age (± standard deviation) of 11.2 ± 3.6 years. Patients were referred to the university clinic for dental examination by the regional thalassemic center. Control group of 198 healthy subjects from the same community tested in a previously validated study (14) were used. Ethical approval for the study was obtained from the Research Committee of Jordan University of Science and Technology, and consent was obtained from parents of all participants. Family history revealed that 41% of the patients were the product of first-cousin marriage, 32% of second-degree cousins, and 27% of distally related or not related. Consanguineous marriage in Jordan showed that first cousin marriage was encountered in 32%, second cousin in 6.8%, distant relation in 10.5%, and no relation in 50% (15).

Alginate impressions were taken in suitable perforated trays for the upper and lower dental arches of every subject, and cast immediately in dental stone. Teeth were selected for measurements only if they were fully erupted, not noticeably affected by attrition or caries, had not been restored and did not display abnormal crown morphology. MD was registered for each maxillary and mandibular permanent tooth from the first molar on one side to the corresponding tooth on the contralateral side. The MD of a tooth obtained by measuring the greatest distance between proximal surfaces of the crown using electronic digital sliding caliper to the nearest 0.01 mm (Mitutoya Co., Utsunomiya, Japan). Intra-observer reliability or precision (differences between the repeated measurements) and inter-observer errors (differences between the means of two sets measurements) were 0.08 mm and 0.16 mm, respectively; representing only 1.2% and 2.1% of the mean measurements.

Sexual dimorphism in tooth crown size was quantified by expressing the percent to which the crown diameters of males exceeded those of females for each individual as follow: 100 (male mean divided by the female mean minus1) reported by Garn et al (16). The cumulative MD (total dentition size) was calculated as the sum of the crown size of individual teeth in each arch up to and including the first permanent molars.

In order to evaluate the relationship (discrepancy) between tooth size and occlusion, the overall and anterior tooth size ratios were calculated according to Bolton formula as follow: the overall tooth size ratio = 100 (sum MD of the 12 mandibular teeth, first molar to first molar, divided by the sum of MD of the 12 maxillary teeth, first molar to first molar) and should be 91.3 ± 1.91%. The anterior tooth size ratio = 100 (sum MD of the six mandibular anterior teeth, canine to canine, divided by the sum MD of the six maxillary teeth, canine to canine) and should be 77.2 ± 1.65%; suggested by Bolton for ideal occlusal relationship (4).

Descriptive statistics including the mean of MD, standard deviation (SD), standard error of the mean (SEM), and coefficient of variation (CV=100 SD / mean) were computed for each individual tooth. Pearson’s correlation coefficient (r) was used to express the degree of association among pairs of homologous (antimeric) teeth. Statistical analyses were done using analysis of variance (ANOVA) and Student t-test to evaluate differences between parameters tested. The level of significance was set as P<0.05.

## Results

There were no significant differences in both sexes between the MD of teeth on the right and left sides of the dental arch. The differences ranged from 0.00 to 0.18 mm, with the greatest differences representing 2.25% of the mean measurements. The correlation coefficient between homologous teeth ranged from 0.74 to 0.84.  Table 1 provides the mean MD for each individual tooth in males and females thalassemic group and the unaffected control group. In both groups, all means for crown size in males exceeded those in females; with the greatest sexual differences being found in the molars (mean = 0.43 mm) followed by canines (mean = 0.38 mm). In 19 of the 24 comparisons, males showed significantly larger MD than females ranged from P<0.05 to P<0.001. The teeth which showed no significant differences between thalassemic males and females were the maxillary and mandibular premolars, whilst in the control group were the mandibular central incisors. In absolute term, thalassemic males had larger MD than females with differences varied from 0.12 mm in the mandibular second premolars to 0.48 mm in the maxillary first molars; weighted differences 0.28 mm on average.

**Table 1:**
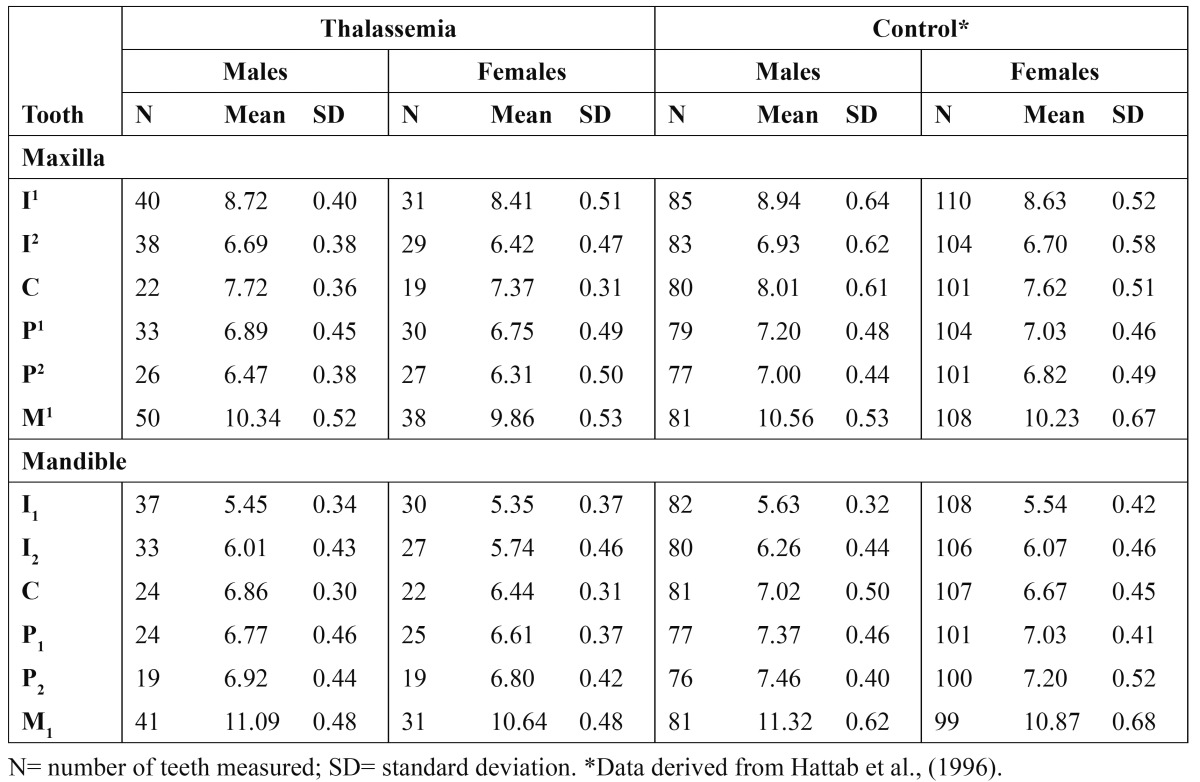
Mesiodistal crown diameters (in mm) of the permanent teeth in thalassemic subjects and control group (right- and left-side measurements pooled).

All means for MD in thalassemic males and females were smaller than the controls, with 20 of the 24 compari-sons being statistically significant ( Table 2, Table 3). The MD in thalassemic group was reduced by an average of 0.31 mm compared with the control group.

**Table 2:**
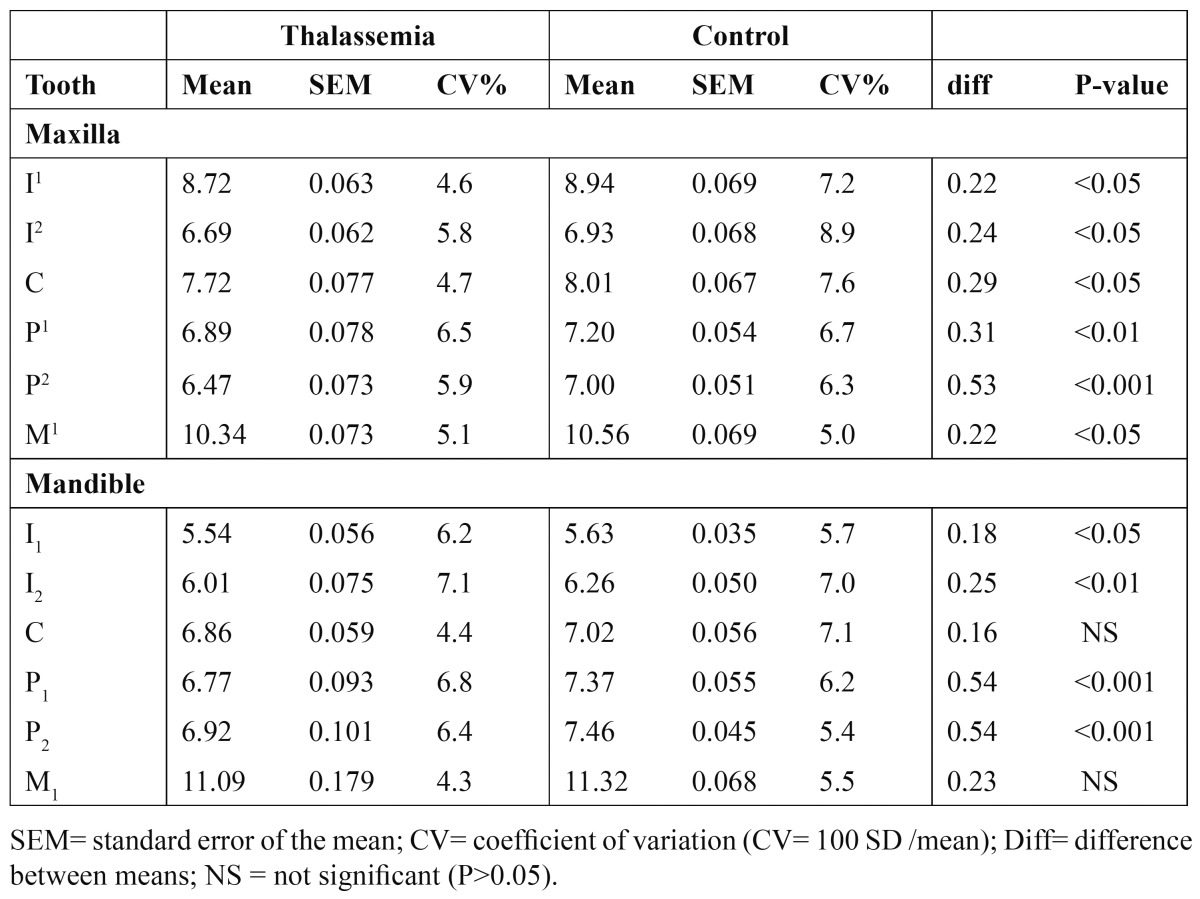
Mesiodistal crown diameters (in mm) of permanent teeth in thalassemic males compared with their counterparts of the control group.

**Table 3:**
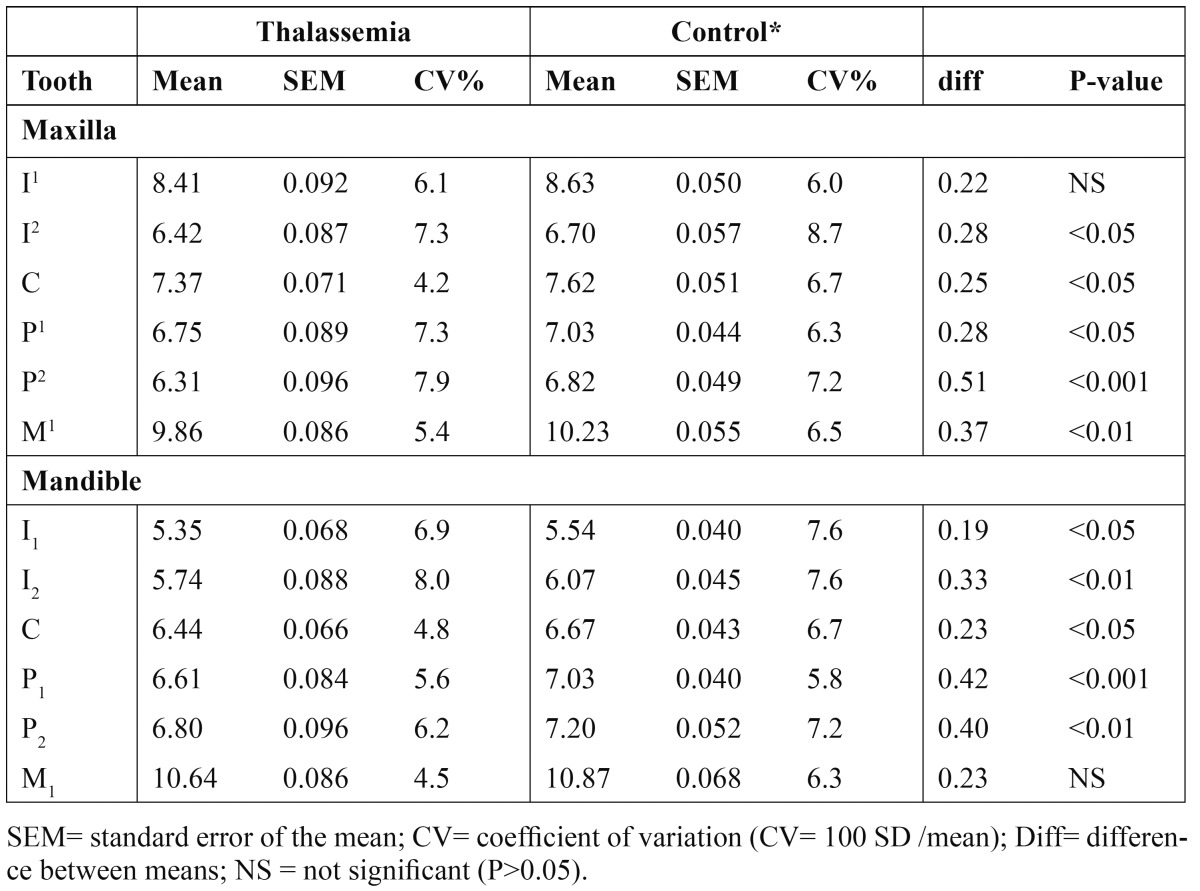
Mesiodistal crown diameters (in mm) of permanent teeth in thalassemic females compare with their counterparts of the control group.

The relative variability in MD indicated by the coefficient of variation (CV) for males and females in thalassemic and control groups are shown in  Table 2, Table 3. Measurements showed that variability differed between individual tooth, with the lateral incisors exhibited the greatest variability in both thalassemic and control groups (CV=7.6%) whilst the first molars displayed the least variable teeth (CV=5.3%). Only slight differences in the mean variability were found between males and females (CV= 6.1% vs. 6.5%) and between maxillary and mandibular teeth (CV = 6.4% vs. 6.2%).

Sexual dimorphism percentages in MD of thalassemic and control groups were similar (3.7% vs. 3.6%). Ranking sexual dimorphism according to the morphological classes revealed the following order: canine (5.4%) > molars (4.1%) > incisors (3.3%) > premolars (2.9%). Similar ranking order of morphological classes was found in the control group. The mandibular central incisors exhibited the least (1.7%), while the mandibular canines (5.9%) displayed the greatest dimorphism in the dentition.

The cumulative MD of the maxillary and mandibular teeth in thalassemic and control groups is presented in  Table 4. In thalassemic males and females the cumulative MD of the maxillary teeth exceeded those of the mandibular teeth in average of 7.5 mm and 7.1 mm, respectively. The corresponding values in the control group were 7.1 mm and 7.4 mm. In both maxillary and mandibular teeth the cumulative MD in thalassemic group were significantly smaller than controls (P<0.001). Calculation of tooth-size Bolton ratios (in percentage) are presented in  Table 4. The anterior and overall ratios (sexes pooled) in thalassemic group were 79.1 and 92.0, respectively. The corresponding ratios of the control group were 79.4 and 92.4, respectively. There were no statistically significant differences in the anterior and overall ratios between sexes or thalassemic and control groups. The anterior and overall ratios in both thalassemic and control groups were significantly larger than Bolton ratios at the level of P<0.001 and P<0.05, respectively ( Table 4).

**Table 4:**
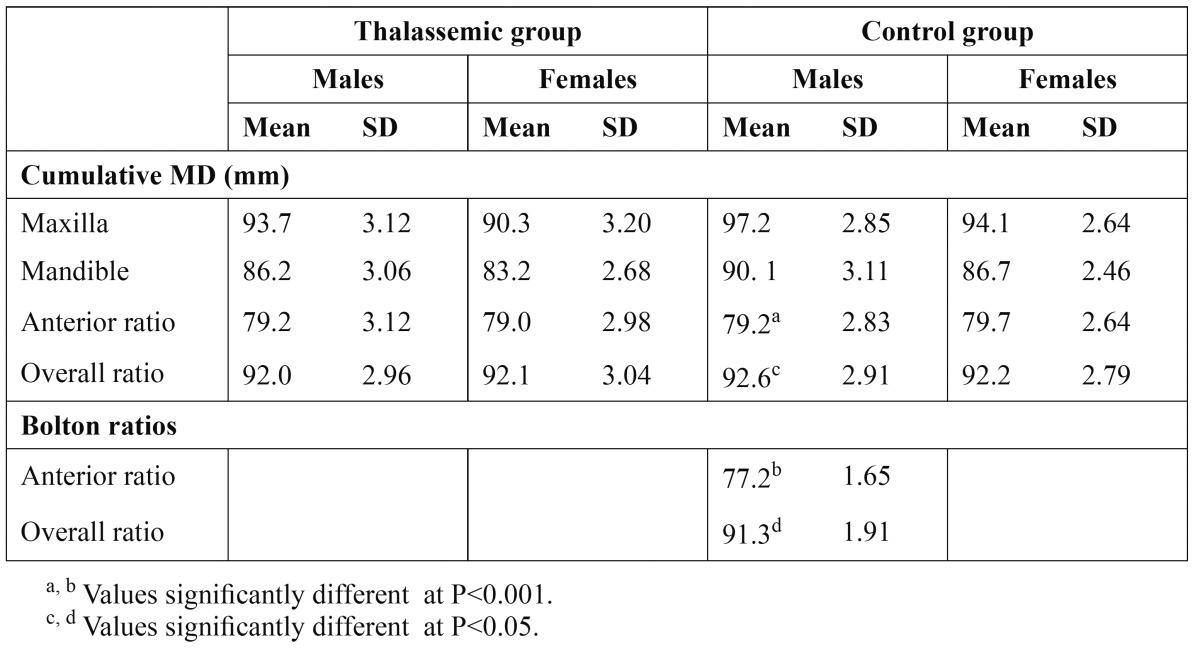
Cumulative mesiodistal crown diameters (MD) and percentage anterior and overall tooth-size discrepancy (TSD) in the thalassemic and control groups compared with Bolton norm.

## Discussion 

Evidence indicates that tooth size exhibits a continuous range of variation among individuals and between different populations (14), a reflection of complex interaction between a variety of genetic and environmental factors.

Tooth crown dimensions are influenced by various maternal conditions and gestational variables including diabetes, hypothyroidism, hypertension, birth weight and birth length (17). Reduction of tooth size was found in Down’s syndrome, oligodontia, and cleft lip and palate patients (18-20). It has been demonstrated that dental development is less variable than skeletal maturation. Greater delays in skeletal maturation than dental development have been found in cases of hypopituitarism, short familial stature, and cerebral palsy (21,22).

The present findings showed that means for MD in the thalassemic group were significantly smaller than the control group. Males in thalassemic and control groups exhibited larger crown size than those of females, the largest differences in both groups being found in the molars and canines. Sexual dimorphism percentages showed that the canines are the most sexual dimorphic teeth in the dentition. The overall percentage of sexual dimorphism in the present sample (3.6%) is similar to that reported for North American whites (3.7%) (23) but somewhat greater than those found in Swedish (3.1%) (24) and Japanese (3.2%) (25) samples. The present study and those reported previously showed that the lateral incisors in both deciduous and permanent dentition were the most variable teeth in the MD, while the first molars in the permanent dentition and the second molars in the deciduous dentition were the most stable teeth (23-25).

Accumulated evidence demonstrated that the MD of the maxillary and mandibular teeth should match each other for a balanced occlusion.  Table 4 present the sum of the maxillary and mandibular MD in both thalassemic and control groups as well as tooth-size ratios. Calculation of the percentage tooth-size ratios showed that the mean anterior and overall ratios in both thalassemic and control groups were significantly larger than Bolton’s standards for ideal occlusion. A review of literature showed that most studies did not find appreciable differences in the mean tooth-size ratios between sexes but significant differences in the ratios exists among various populations with anterior ratio ranged between 77.2 and 81.5 and overall ratio between 89.9 and 93.1 (26) and among orthodontic patients (27). In clinical practice, attention should be paid to TSD between the maxillary and mandibular teeth for orthodontic diagnosis and treatment planning that would improve achieving optimal occlusion, overbite and overjet. It seems necessary to determine specific standards of tooth-size ratios for different populations since the ideal relationships established by Bolton for American whites are not always applicable for other racial groups.

## References

[1] Radnzic D (1988). Dental crowding and its relationship to mesiodistal crown diameters and arch dimensions. Am J Dentofac Orthop.

[2] Forsberg CM (1988). Tooth size, spacing and crowding in relation to eruption or impaction of third molars. Am J Dentofac Orthop.

[3] Hattab FN, Abu Alhaija ESJ (1999). Radiographic evaluation of mandibular third molar eruption space. Oral Surg Oral Med Oral Pathol Oral Radiol Endod.

[4] Bolton A (1962). The clinical application of a tooth-size analysis. Am J Orthod.

[5] Olivieri N (1999). The β-thalassemias. N Engl J Med.

[6] Modell CB (1974). The pathophysiology of beta-thalassaemia major. J Clin Pathol.

[7] Van Dis ML, Langlais RP (1986). The thalassemias: Oral manifestations and complications. Oral Surg Oral Med Oral Pathol.

[8] Abu Alhaija ESJ, Hattab FN, al-Omari MA (2002). Cephalometric measurements and facial deformities in subjects with β-thalassemia major. Eur J Orthod.

[9] Hattab FN (2012). Periodontal condition and orofacial changes in patients with thalassemia major: a clinical and radiographic overview. J Clin Pediatr Dent.

[10] Leonardi R, Verzi P, Caltabiano M (1990). Epidemiological survey of the prevalence of dental caries in young thalassemia major patients. Stomatol Mediterr.

[11] Hattab FN, Hazza'a AM, Yassin OM, AL-Rimawi HS (2001). Caries risk in patients with thalassemia major. Int Dent J.

[12] Hattab FN, Yassin OM (2011). Dental arch dimensions in subjects with β-thalassemia major. J Contemp Dent Pract.

[13] Hattab FN (2013). Patterns of physical growth and dental development in Jordanian children and adolescents with thalassemia major. J Oral Sci.

[14] Hattab FN, Al-Kateeb S, Sultan I (1996). Mesiodistal crown diameters of permanent teeth in Jordanians. Archs oral Biol.

[15] Khoury SA, Massad D (1992). Consanguineous marriage in Jordan. Am J Med Genet.

[16] Garn SM, Lewis AB, Swindler DR, Kerewsky RS (1967). Genetic control of sexual dimorphism in tooth size. J Dent Res.

[17] Garn SM, Osborne RH, McCabe KD (1979). The effect of prenatal on crown dimensions. Am J Phys Anthrop.

[18] Townsend GC (1983). Tooth size in children and young adults with trisomy 21 (Down) syndrome. Arch oral Biol.

[19] van der Weide YS, Steen WHA, Beemer FA, Bosman F (1994). Reductions in size and left-right asymmetry of teeth in human oligodontia. Arch oral Biol.

[20] Rawashdeh MA, Bakir IFB (2007). The crown size and sexual dimorphism of permanent teeth in Jordanian cleft lip and palate patients. Cleft Palate-Craniofac J.

[21] Lewis AB, Garn SM (1960). The relation between tooth formation and other maturational factors. Angle Orthod.

[22] Cardoso HF (2007). Environmental effects on skeletal versus dental development: Using a documented subadult skeletal sample to test a basic assumption in human osteological research. Am J Phys Anthropol.

[23] Moorrees CFA, Thomsen SO, Jensen E, Yen PKJ (1957). Mesiodistal crown diameters of deciduous and permanent teeth in individuals. J Dent Res.

[24] Lysell L, Myrberg N (1982). Mesiodistal tooth size in the deciduous and permanent dentitions. Eur J Orthod.

[25] Ooshima T, Ishida R, Mishima K, Sobue S (1996). The prevalence and developmental anomalies of teeth and their association with tooth size in the primary and permanent dentitions of 1650 Japanese children. Int J Paediatr Dent.

[26] Al-Omari IK, Al-Bitar ZB, Hamdan AM (2008). Tooth size discrepancies among Jordanian schoolchildren. Eur J Orthod.

[27] Wedrychowska-Szulc B, Janiszewska-Olszowska J, Stepien P (2010). Overall and anterior Bolton ratio in Class I, II, and IIIorthodontic patients. Eur J Orthod.

